# Clinicopathological Characteristics, Molecular Findings, and Survival of Lung Cancer in Adults Aged <30 Years in China: A Retrospective Cohort Study

**DOI:** 10.3390/jcm15166360

**Published:** 2026-08-18

**Authors:** Jingchen Zhang, Jianya Zhou

**Affiliations:** Department of Respiratory Disease, Thoracic Disease Centre, The First Affiliated Hospital, College of Medicine, Zhejiang University, Hangzhou 310003, China; 1515001@zju.edu.cn

**Keywords:** lung neoplasms, young adult, carcinoma, mutation, survival analysis

## Abstract

**Background/Objectives:** Lung cancer is increasingly recognized in young adults, but data specific to patients younger than 30 years remain scarce. We aimed to characterise the clinicopathological features, molecular profiles, and survival outcomes of lung cancer in adults younger than 30 years. **Methods:** A retrospective cohort study was conducted at the Department of Respiratory Disease, The First Affiliated Hospital, College of Medicine, Zhejiang University, Hangzhou, China. Patients aged 18 to 30 years with pathologically confirmed primary lung cancer, admitted between August 2011 and October 2021, were identified from the pathology and oncology databases of the study centre; eligibility and exclusion criteria and the resulting analytic cohort are summarized below (a screened/excluded patient count could not be reconstructed retrospectively; see Limitations). Demographics, histology, stage (eighth-edition TNM), driver mutations, and treatment were extracted, and the extent of missing data for each variable was assessed. Molecular testing was not performed prospectively for this study; rather, we report driver-mutation results that had already been generated as part of routine clinical care for patients in whom testing was clinically indicated and archived tissue was available. Overall survival (OS) was estimated using the Kaplan–Meier method and compared using the log-rank test; hazard ratios (HRs) were derived from univariate Cox regression and are reported as exploratory/descriptive given the non-randomized comparison (see Discussion). **Results:** Among 139 patients meeting eligibility criteria, the cohort was predominantly female (65.5%) and most individuals were never-smokers (92.8%), with adenocarcinoma predominating (93.5%). Stage I (61.2%) and stage IV (30.2%) were most common. Of 34 patients (24.5%) with available molecular testing results, 23 (67.6%) carried a driver mutation, of whom 19 (82.6%) had stage IV disease. Among mutation-positive patients—drawn from a selectively tested subcohort enriched for advanced-stage disease—ALK rearrangement was most frequent (13; 56.5%). In the stage IV subgroup, patients who received first-line tyrosine kinase inhibitors (TKIs) had a longer observed OS than those who did not (median, 88 vs. 24 months); this exploratory, non-randomized comparison is reported descriptively rather than as an adjusted treatment-effect estimate (see Limitations). **Conclusions:** In this single-centre retrospective cohort, adults aged <30 years with lung cancer were predominantly female never-smokers with adenocarcinoma. Among the selectively tested advanced-stage subgroup, ALK fusions were common. These data justify further multicentre study and broader contemporary molecular characterisation.

## 1. Introduction

Lung cancer remains the leading cause of cancer-related morbidity and mortality worldwide. According to the GLOBOCAN 2022 estimates, lung cancer was the most frequently diagnosed cancer globally, with approximately 2.48 million new cases (12.4% of all cancers) and 1.82 million deaths (18.7% of all cancer deaths), making it both the most common cancer and the leading cause of cancer death worldwide [1]. In China specifically, lung cancer alone accounted for an estimated 1.06 million new cases and 0.73 million deaths in 2022, ranking first among all cancer types in both incidence and mortality nationally; lung cancer was also the single largest contributor among the five leading cancer types (lung, colon-rectum, thyroid, liver, and stomach) that together accounted for approximately 57% of the 4.82 million new cancer cases estimated in China that year [2]. Although advanced age is a well-established risk factor, recent data show an increasing incidence of early-onset lung cancer among young adults, particularly in China [3]. Lung cancer in younger Chinese patients (aged <45 years) currently accounts for approximately 3.5% of the total disease burden [4].

Clinical characteristics, molecular profiles, demographic features, and treatment responses differ substantially between younger (aged <45 years) and older patients, as highlighted in recent reviews and population-based analyses of lung cancer in adolescents and young adults [5]. Available evidence indicates a predominance of female patients, never-smokers, and adenocarcinoma histology in young patients [6]. In addition, oncogenic driver mutations, such as those in anaplastic lymphoma kinase (ALK), epidermal growth factor receptor (EGFR), and ROS1, are reported more frequently in early-onset than in late-onset disease [7,8,9]. Nevertheless, studies focusing specifically on patients younger than 30 years are scarce, and the reported findings vary widely. The genomic landscape and long-term prognosis of Chinese patients younger than 30 years with lung cancer remain poorly characterized.

We selected 30 years as the upper age boundary for three reasons. First, most published series of “young” lung cancer use age cut-offs of 40 or 45 years [4,5]; within these broad bands, patients in their late thirties or early forties dominate numerically and can obscure the clinicopathological and molecular profile of the most extreme age group, in whom heritable or early-life carcinogenic mechanisms are more likely to be biologically relevant than cumulative environmental exposure. Restricting the cohort to patients younger than 30 years isolates this more homogeneous, biologically distinct extreme of the age spectrum. Second, 30 years approximates the upper limit used by several prior single-institution reports of the youngest lung cancer patients in China and elsewhere [9,10,11], allowing our findings to be compared directly with that literature. Third, from a clinical-service perspective, patients under 30 years old are typically still completing education or establishing careers and families, so the treatment-decision context, tolerance for treatment-related morbidity, and long-term survivorship priorities differ from those of patients in their forties; characterizing this narrower group separately has direct relevance for counselling and treatment planning.

Because current data on lung cancer in patients younger than 30 years come from a small number of cohorts, contemporary demographic trends, treatment efficacy, and clinical outcomes in this population warrant further study. We therefore retrospectively evaluated the clinicopathological characteristics, molecular profiles, and long-term survival outcomes of patients aged 18 to 30 years with lung cancer treated at The First Affiliated Hospital, College of Medicine, Zhejiang University. These real-world data may help inform precision treatment and improve outcomes in this population.

## 2. Materials and Methods

### 2.1. Study Design, Eligibility Criteria, and Patient Flow

This retrospective cohort study was designed and is reported in accordance with the STROBE (Strengthening the Reporting of Observational Studies in Epidemiology) statement for cohort studies. Clinical data were retrospectively extracted from the electronic medical record system of The First Affiliated Hospital, College of Medicine, Zhejiang University, over a 10-year enrolment period (August 2011 to October 2021); this 10-year window describes the period during which patients were admitted and identified for the cohort, not a uniform 10-year follow-up duration for each patient. Because patients entered the cohort at different calendar times across this window, individual follow-up duration necessarily varies (from a minimum of approximately 4 years for patients admitted at the end of the enrolment window to over 14 years for patients admitted at the start), and follow-up length is summarized descriptively in Section 2.6 and Section 3.4 rather than assumed to be 10 years for every patient.

Patients were identified directly from the pathology and oncology databases of the Department of Respiratory Disease and Thoracic Surgery by searching for a pathological diagnosis of primary lung malignancy in patients aged 18 to 30 years at admission; this pathology-anchored search strategy, rather than a prospective screening log of all young patients presenting with a lung lesion, was used throughout the 10-year study period. Patients were retained in the study cohort if they met both of the following inclusion criteria: (i) pathologically confirmed primary lung malignancy, established by biopsy, surgical resection, or cytology; (ii) age of 18 to 30 years (inclusive) at initial admission to our centre. Patients with adenocarcinoma in situ (AIS) were included and analyzed as a separate subset because of its distinct clinical course in this age group.

Patients were excluded if they had any of the following: (i) secondary or metastatic tumors arising from another primary organ; (ii) haematologic malignancy involving the lung parenchyma; or (iii) insufficient clinical or pathological data for baseline evaluation. These inclusion and exclusion criteria are summarized in Table 1. After exclusions, 139 patients constituted the analytic cohort. Because our search strategy began from a confirmed pathological diagnosis rather than from a prospectively logged pool of all young patients evaluated for a lung lesion (including those ultimately found not to have a primary lung malignancy), an aggregate count of patients screened and excluded at each step, as conventionally displayed in a STROBE flow diagram, could not be reconstructed retrospectively from the source pathology and oncology databases; no such screening log was maintained at the time of data collection. We report this explicitly as a limitation of the retrospective design (Section 4) rather than estimate these figures.

**Table 1:** Inclusion and Exclusion Criteria.

Criteria	Details
Inclusion criteria	(i) Pathologically confirmed primary lung malignancy, established by biopsy, surgical resection, or cytology; (ii) age of 18 to 30 years (inclusive) at initial admission to the study centre from August 2011 to October 2021. Adenocarcinoma in situ (AIS) was included and analyzed as a separate subset (Section 2.1).
Exclusion criteria	(i) Secondary or metastatic tumors arising from another primary organ; (ii) haematologic malignancy involving the lung parenchyma; (iii) insufficient clinical or pathological data for baseline evaluation (Section 2.1).
Note on exclusion counts	Because patients were identified via a pathology-anchored database search rather than a prospectively logged screening registry of all young patients evaluated for a lung lesion, an aggregate screened/excluded patient count is not available for this retrospective cohort (Section 2.1 and Section 4). The criteria above are nonetheless applied uniformly and are also depicted graphically in Figure 1.

**Figure 1:**
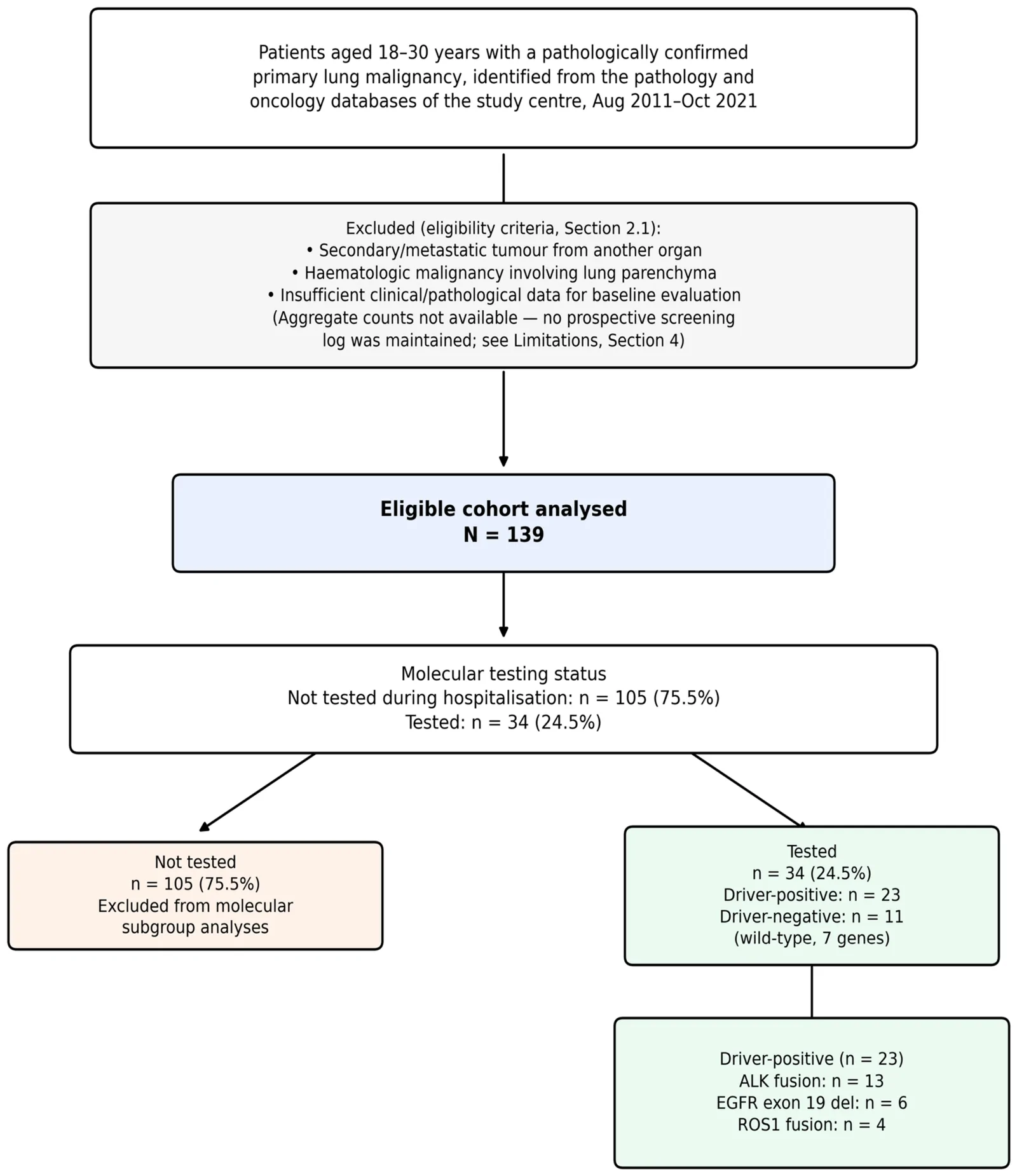
Patient identification, eligibility, and molecular testing status. Patients were identified from the pathology and oncology databases of the study centre by searching for a pathological diagnosis of primary lung malignancy in patients aged 18–30 years at admission between August 2011 and October 2021 (Section 2.1); this pathology-anchored ascertainment method, rather than a prospectively logged screening registry of all young patients evaluated for a lung lesion, was used throughout the study period, so the total number initially screened and the number excluded at each step cannot be reported (Section 4). After applying the eligibility and exclusion criteria in Section 2.1, 139 patients constituted the analytic cohort. Of these, 34 (24.5%) had molecular testing results available from routine clinical care (Section 2.4), yielding 23 driver-positive and 11 driver-negative patients.

This table summarizes the eligibility and exclusion criteria applied to assemble the 139-patient analytic cohort; a corresponding graphical version is provided in Figure 1.

The variables collected included baseline demographics (age and sex), smoking history, and clinical presentation (symptomatic versus incidentally detected on imaging, where recorded). Pathological and morphological characteristics recorded included histologic subtype (adenocarcinoma, squamous cell carcinoma, small cell carcinoma, or other, as detailed further in Section 2.2 and Section 3.1), tumor laterality and lobar location, and TNM stage (eighth edition, Section 2.2); tumor differentiation grade and detailed adenocarcinoma growth-pattern subtyping (e.g., lepidic, acinar, papillary, micropapillary, or solid predominant) were not uniformly recorded across the 10-year (2011–2021) collection period in a form that could be extracted retrospectively for all patients, and are therefore not reported as a separate variable in this analysis; we note this as an area for more detailed prospective characterization in future cohorts. Molecular profile (driver gene mutations, Section 2.4), treatment (Section 3.3), and baseline performance status when available (Section 2.5) were also recorded. Smoking status was classified as never-smoker or ever-smoker (current or former). The study was approved by the Institutional Ethics Committee of The First Affiliated Hospital, College of Medicine, Zhejiang University (protocol code 20250032; approved 23 May 2025). Because retrospective chart review at our centre spanned a 10-year historical window (2011–2021) during which some patients were enrolled as minors under local statute at the time of their initial diagnosis and tissue banking, written informed consent for the research use of clinical data and archived tumor tissue was obtained from each patient if aged 18 years or older at the time consent was sought, or from the patient’s legal guardian if the patient was younger than 18 years at that time; all patients included in the final analytic cohort (18–30 years at admission) were themselves legally adults in China (age of majority, 18 years), and guardian consent applied only to the small number of historical cases in which initial tissue archiving or an earlier consent process occurred before the patient turned 18. This is now stated explicitly to avoid the impression that adult patients required guardian consent.

### 2.2. Histologic Classification and Terminology

For clarity, we specify our use of histologic terminology here. Non-small cell lung cancer (NSCLC) is used throughout this manuscript as an umbrella category comprising all lung cancer histologic subtypes other than small cell carcinoma; in our cohort, NSCLC therefore includes adenocarcinoma (the predominant subtype, Section 3.1), squamous cell carcinoma, and the miscellaneous subtypes. Adenocarcinoma is accordingly a specific histologic subtype within the broader NSCLC category, not a synonym for it, and we use “adenocarcinoma” only when referring specifically to that subtype (e.g., in the mutation-testing and treatment sections, where testing and targeted therapy were concentrated in adenocarcinoma). Adenocarcinoma in situ (AIS) is classified separately from invasive adenocarcinoma and is reported as its own category throughout, consistent with its distinct clinical behaviour (Section 2.1).

### 2.3. Staging

To ensure consistency across the 10-year study period (2011–2021), all cases were staged according to the eighth edition of the tumor-node-metastasis (TNM) classification. For cases originally staged with the seventh edition, two senior thoracic oncologists independently reviewed the original records, surgical notes, and imaging—including chest computed tomography (CT), cranial magnetic resonance imaging (MRI), and positron emission tomography/CT (PET/CT) when available—and restaged each case according to the eighth edition to minimize misclassification.

### 2.4. Driver Gene Mutation Assessment

Molecular testing in this cohort was not performed prospectively for the purposes of this study. Rather, we retrospectively collected driver-mutation results that had already been generated during routine clinical care, where testing was ordered by the treating physician when clinically indicated and archived tumor tissue or cytology specimens were available in sufficient quantity and quality. Testing was therefore opportunistic rather than systematic, and eligibility for testing in real-world practice was influenced by disease stage, histologic subtype, tissue adequacy, patient/family preference, and reimbursement policy, all of which changed over the 10-year study period, as detailed below.

Institutional and national testing practice evolved substantially between 2011 and 2021. In the earlier years of the study period, molecular testing at our centre was performed selectively, largely restricted to patients with advanced-stage (stage IIIB/IIIC/IV) non-squamous non-small cell lung cancer (NSCLC) for whom targeted therapy was being considered, consistent with the Chinese Society of Clinical Oncology (CSCO) and national reimbursement guidelines in effect at the time, which did not recommend routine molecular testing for early-stage, completely resected disease. From approximately 2015 onward, as next-generation sequencing (NGS) became more widely available and additional targeted agents were approved in China, testing was increasingly extended to node-positive resected disease (stage II-IIIA) and, in the final years of the study period, offered more broadly regardless of stage when tissue was available, although uptake in early-stage disease remained low relative to advanced-stage disease because it was not mandated by guidelines or reimbursement criteria for stage I disease. Consequently, the 34 patients with available results in this cohort are not a random or complete sample of the 139 eligible patients; they represent the subset for whom testing was clinically ordered and technically feasible, and are enriched for advanced-stage, non-squamous adenocarcinoma, as quantified in Section 3.1.

Targetable alterations—EGFR mutations, KRAS mutations, BRAF V600E, HER2 insertions, MET exon 14 skipping, ALK fusions, and ROS1 translocations—were evaluated by polymerase chain reaction (PCR)-based pyrosequencing (used predominantly in the earlier study years for EGFR hotspot mutations) or next-generation sequencing (NGS) targeted panels (increasingly used from approximately 2015 onward, encompassing the seven genes listed above). Positive ALK results were confirmed by fluorescence in situ hybridization (FISH) or immunohistochemistry (IHC) with the D5F3 antibody. Because assay platforms changed over the study period, we did not perform a single uniform panel across all 34 tested patients; the specific method used for each patient corresponded to the standard-of-care assay available at our centre at the time of that patient’s testing. This heterogeneity in testing era, indication, and platform is a limitation discussed in Section 4.

### 2.5. Missing Data

As in most retrospective cohorts spanning a decade, some variables were incompletely documented in the medical record, particularly for patients treated earlier in the study period. We handled missing data as follows. For descriptive analyses, the denominator for each variable is the number of patients with a recorded value for that variable (complete-case analysis); missing observations were not imputed, and percentages are reported relative to the available (non-missing) denominator unless otherwise specified. Missing baseline variables were not carried forward or collapsed into an “unknown” category within statistical comparisons; instead, patients with missing values for a given variable were excluded from analyses involving that specific variable, and the applicable denominator is stated in the corresponding table or text. The extent of missingness for each variable used in the descriptive and survival analyses is summarized qualitatively in Table 2.

Two variables were particularly affected: Eastern Cooperative Oncology Group (ECOG) performance status and detailed metastatic burden (number and site of metastases at diagnosis in stage IV patients). Both were recorded inconsistently in free-text clinical notes rather than in a structured, mandatory field of the electronic medical record during the earlier years of the study period, and neither was captured in the structured dataset originally extracted for this study; we are therefore unable to report an exact denominator or percentage missing for these two variables; a full re-abstraction of free-text notes for all 139 patients was beyond the scope of this revision. We consider this an important limitation of the retrospective design rather than a gap that can be closed by re-analysis, and it is the principal reason we did not perform multivariable adjustment or propensity-score matching for the stage IV treatment comparison, presenting it instead as an exploratory, descriptive analysis (Section 2.7 and Section 3.4). Family history of malignancy and passive-smoking/cooking-oil fume exposure were similarly documented only sporadically in free text and are discussed narratively rather than quantitatively (Section 4); no multiple-imputation analysis was attempted for any of these variables, and we regard prospective, structured capture of performance status and exposure history as a priority for the multicentre follow-up study we propose.

### 2.6. Follow-Up

Follow-up was conducted through outpatient visits, structured telephone interviews, and review of medical records. The follow-up period extended from the date of initial pathological diagnosis to the study cutoff of 31 December 2025; follow-up was deliberately extended beyond the enrolment window to allow sufficient maturity of survival data, given the prolonged survival expected in this young, predominantly driver-positive population. OS was defined as the interval from initial pathological diagnosis to death from any cause or the last verified contact. Patients lost to follow-up were censored at the date of last contact. Median follow-up among survivors was calculated using the reverse Kaplan–Meier method.

### 2.7. Statistical Analysis

Survival curves were estimated using the Kaplan–Meier method, and groups were compared using the log-rank (Mantel–Cox) test. The Kaplan–Meier method explicitly accommodates patients with unequal follow-up duration through right censoring: patients who were event-free at their last contact (including those who entered the cohort more recently and therefore have shorter potential follow-up, given the 10-year, 2011–2021 enrolment window described in Section 2.1) contribute survival time up to that point and are then censored, rather than being treated as equivalent to patients with a full observation period; this is the standard and appropriate approach for cohorts with staggered entry and is why we additionally report the number of patients at risk at each time point in the corresponding survival curves, so that readers can judge how many patients actually contributed follow-up data at longer time points. Baseline characteristics of the molecularly tested and untested groups were compared with the χ^2^ test or the Fisher exact test to assess testing bias. We considered a multivariable Cox regression across the whole cohort (e.g., incorporating age, sex, stage, histology, and driver-mutation status as covariates for overall survival) but concluded this was not appropriate given the small number of outcome events relative to the number of candidate covariates, particularly outside stage III/IV disease where median OS was not reached for AIS or stage I disease (Section 3.4) and few deaths had occurred at the time of analysis; standard guidance for Cox regression recommends at least 10 events per covariate, a threshold this cohort’s early-stage event count does not support. In addition, ECOG performance status could not be reliably captured for the whole cohort (Section 2.5); a model built under these constraints would be at high risk of overfitting and its coefficients would not be reliably interpretable. We therefore restricted regression-based comparison to the univariate, hypothesis-generating stage IV analysis described below, and present the whole-cohort survival differences by stage descriptively via Kaplan–Meier curves, with accompanying numbers at risk. For the stage IV treatment comparison specifically, HRs and 95% CIs were estimated using a univariate Cox proportional hazards model; the proportional hazards assumption was tested using Schoenfeld residuals. We explicitly reframe this comparison as exploratory and descriptive rather than as an adjusted estimate of treatment effect, for the following reasons, detailed further in Section 3.4 and Section 4: the non-targeted comparator arm combines driver-negative, untested, and treatment-refusing patients into a single heterogeneous group; baseline ECOG performance status could not be reliably tabulated by subgroup because it was captured only sporadically in free-text notes rather than in a structured field (Section 2.5, Table 2); and because of the small subgroup sizes (*n* = 19 vs. *n* = 23 for the TKI comparison), multivariable adjustment and propensity-score matching were not feasible. Where the underlying subgroup counts are small, we report the HR alongside the descriptive median OS values but interpret the difference as a longer observed OS in the subgroup that received first-line TKIs rather than as a causal treatment effect. Numbers at risk were calculated for each survival analysis. All analyses were performed in GraphPad Prism 8.0 (GraphPad Software). A two-sided *p* value < 0.05 was considered statistically significant.

## 3. Results

### 3.1. Patient Flow, Clinicopathological Characteristics, and Comparison by Testing Status

Patient identification, eligibility/exclusion criteria, and the resulting analytic cohort are summarized in Figure 1 (a screened/excluded patient count is not available; see Section 2.1). A total of 139 patients met eligibility criteria and were analyzed. The median age was 26.9 years (range, 18–30 years). A smoking history was uncommon, reported in only 10 male patients (7.2%). Adenocarcinoma was the predominant histologic subtype (93.5%), followed by squamous cell carcinoma (2.9%), small cell lung cancer (1.4%), and miscellaneous histologies (2.2%). Stage I was the most common stage at diagnosis (61.2%), followed by stage IV (30.2%), stage III (3.6%), AIS (2.9%), and stage II (2.2%). Baseline characteristics, together with the number of patients with a recorded (non-missing) value for each variable, are summarized in Table 3. The extent of missing data for each variable used in the descriptive and survival analyses is summarized separately in Table 2.

Because histology and stage were both completely recorded for all 139 patients (Table 2), the joint distribution of these two variables is fully determined by the univariate totals already reported: of the 42 patients with stage IV disease, adenocarcinoma accounted for at least 33 patients, since only 9 patients in the entire cohort had a non-adenocarcinoma histology (4 squamous cell carcinoma, 2 small cell carcinoma, and 3 miscellaneous; Table 3) and, at most, all 9 of these non-adenocarcinoma cases could have been in stage IV. Restricting to the mutation-tested subgroup (Section 2.4), all 34 tested patients had adenocarcinoma histology (Table 3), and 19 of these 34 tested patients had stage IV disease (Section 3.1), so within the tested subgroup the stage IV/adenocarcinoma overlap is precisely 19 patients. A full stage-by-histology cross-tabulation for the untested majority of the cohort was not tabulated in the structured dataset originally extracted for this study; the corresponding author is verifying the exact non-adenocarcinoma count within stage IV against the source pathology records for the next revision cycle, and this will be reported as an exact figure once confirmed.

Genetic testing results were available for 24.5% of patients (34 of 139); as detailed in Section 2.4, this reflects the subset of patients for whom testing was clinically ordered during routine care and archived tissue was available, not a prospectively planned testing protocol. To assess potential selection bias, we compared baseline characteristics between the tested group (*n* = 34) and the untested group (*n* = 105). Testing was more frequent among patients with stage IV disease (tested, 55.9% vs. untested, 21.9%; *p* < 0.001) and adenocarcinoma histology (tested, 100% vs. untested, 91.4%; *p =* 0.034). Age and sex did not differ significantly between groups (both *p* > 0.05).

### 3.2. Molecular Testing Results

**Figure 2:**
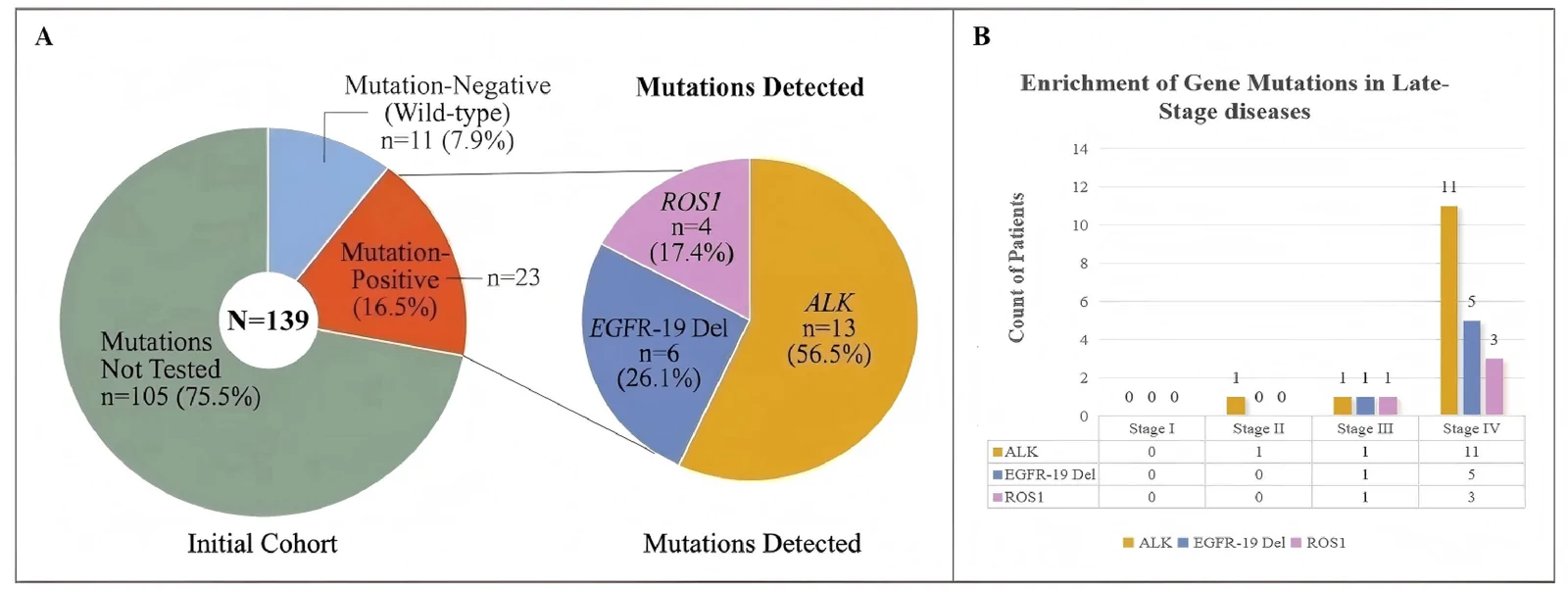
Driver gene mutation landscape among tested patients. (**A**) Breakdown of the cohort (*n* = 139) by testing and mutation status, with the distribution of driver alterations among mutation-positive patients (*n* = 23); because testing was opportunistic and enriched for advanced-stage adenocarcinoma (Section 2.4), this panel characterises the tested subgroup rather than the full cohort. (**B**) Distribution of driver-positive patients by stage (I–IV), showing enrichment of ALK fusion, EGFR exon 19 deletion, and ROS1 fusion in advanced-stage disease. Percentages in panel A are expressed relative to the full cohort (N = 139; e.g., mutation-positive, 23/139 = 16.5%), reflecting the low overall testing rate; among the 34 patients who underwent molecular testing, the driver-positive rate was 67.6% (23/34), as reported in Section 3.1.

Among the 34 tested patients, 23 (67.6%) carried a confirmed driver mutation and 11 (32.4%) were driver-negative. No targetable alterations in KRAS, BRAF, HER2, or MET were detected; all 11 driver-negative patients were wild-type for all seven genes tested. Within the mutation-positive subgroup, ALK fusion was the most frequent alteration (13 patients; 56.5%), followed by EGFR exon 19 deletion (6 patients; 26.1%) and ROS1 fusion (4 patients; 17.4%). Driver-positive status was associated with advanced stage: 19 of the 23 mutation-positive patients (82.6%) had stage IV disease, 3 had stage III disease, and 1 had stage II disease. Mutation-positive patients accounted for 45.2% (19 of 42) of the stage IV subpopulation (here, 42 denotes the total number of stage IV patients in the cohort, not the number of patients tested; this figure should not be confused with the 34 patients who underwent molecular testing, described in Section 3.1). Because these results derive from the selectively tested subcohort described in Section 2.4 and Section 3.1, the mutation frequencies below should be read as characterising the tested, advanced-stage-enriched subgroup rather than the genomic landscape of the full cohort. The molecular distribution is shown in Table 4 and Figure 2.

### 3.3. Treatment

Curative-intent surgical resection was performed in 62.6% of patients (87 of 139). Within this group (*n* = 87), 74 patients received observation alone, 6 received adjuvant chemotherapy, 4 received adjuvant targeted therapy, and 3 received neoadjuvant chemotherapy before resection. Twenty patients in the surgical group also received postoperative thoracic radiotherapy, indicated for pathologically confirmed N2 involvement or positive surgical margins (R1 resection) in accordance with the guidelines in effect at the time.

Of the 21 patients (15.1%) with advanced disease (stage IIIb, IIIc, or IV) who were not candidates for primary resection, 14 received cytotoxic chemotherapy alone and 7 received chemotherapy plus an immune checkpoint inhibitor (ICI). First-line TKI therapy was given to 19 patients with stage IV disease harboring targetable alterations (crizotinib in 16, gefitinib in 2, and alectinib in 1). In addition, 4 mutation-positive patients diagnosed at earlier stages (1 with stage II and 3 with stage III disease) underwent curative resection followed by adjuvant targeted therapy (crizotinib in 1 and gefitinib in 3). Twelve patients (8.6%) declined standard treatment or chose supportive care alone. Treatment allocations are detailed in Table 5.

### 3.4. Survival Outcomes

The follow-up cutoff was 31 December 2025. Nine patients were lost to follow-up (2 with stage I and 7 with stage IV disease) and were censored at last contact. Median follow-up among survivors was 68.5 months (interquartile range, 42.0–98.2 months).

**Figure 3:**
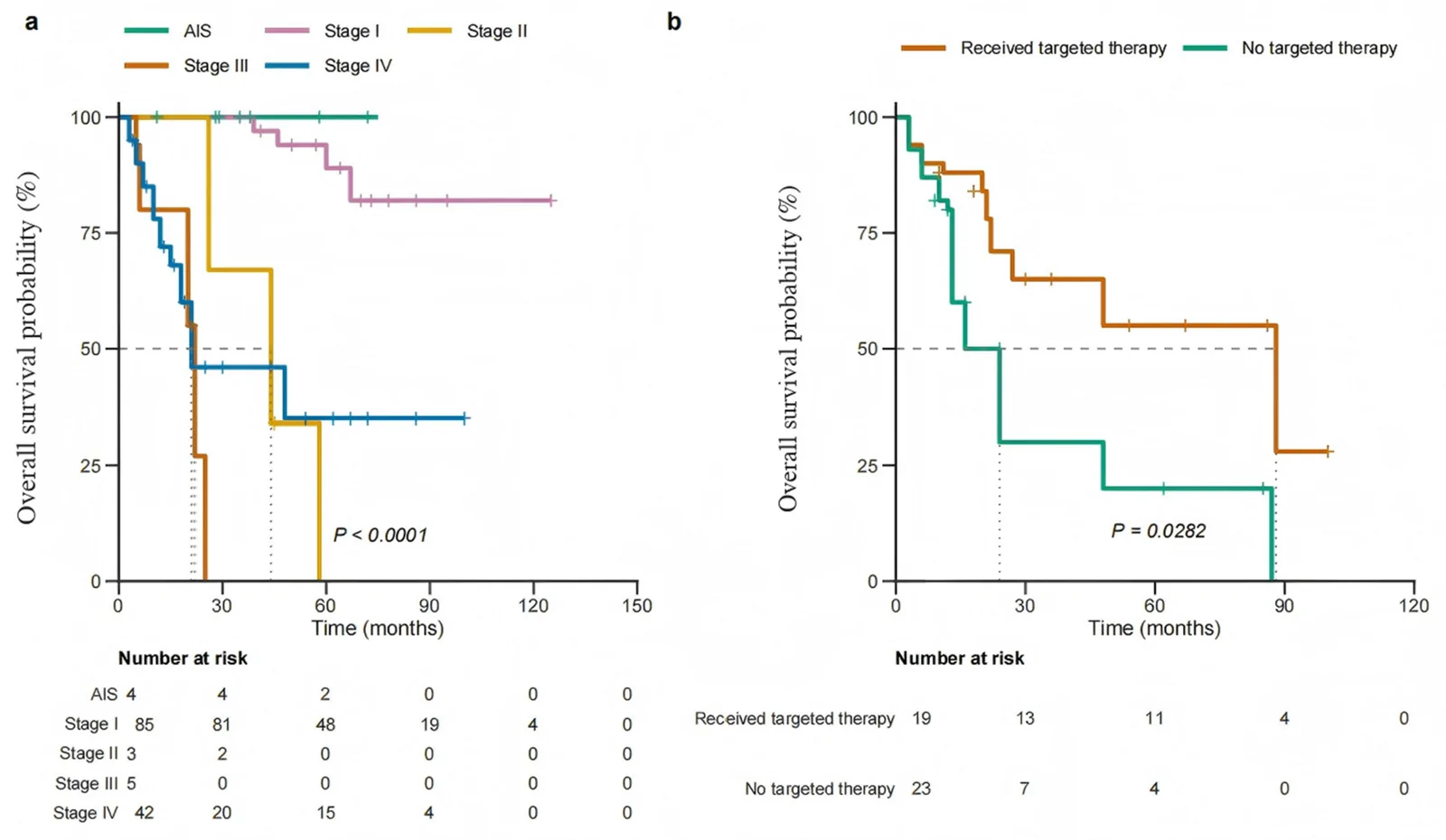
Kaplan–Meier survival analyses. (**a**) OS by stage (eighth-edition TNM), including AIS and stages I to IV NSCLC. The corresponding number-at-risk table is shown to indicate sample-size limitations. The stage II subgroup includes only 3 patients; the abrupt decline in this curve is an artifact of the small sample size and does not represent a true prognosis for this stage, and should be interpreted together with the number-at-risk table. (**b**) OS in patients with stage IV NSCLC, comparing those with targetable mutations who received first-line TKIs with those managed without targeted therapy. This panel is exploratory and descriptive (Section 2.7 and Section 3.4); the comparator arm is heterogeneous and the estimate is not adjusted for baseline confounders. Numbers at risk and log-rank *p* values are shown within each panel.

Median OS was not reached for patients with AIS or stage I disease (survival range, 29–76 and 13–126 months, respectively). Median OS was 44 months (range, 26–58) for stage II, 22 months (range, 12–25) for stage III, and 24 months (range, 2–100) for stage IV disease (Figure 3a). The stage II subgroup comprised only 3 patients (all female never-smokers with adenocarcinoma). Patient 1 (aged 24 years; stage IIB [pT3N0M0]; ALK fusion) received adjuvant targeted therapy but developed rapid multiorgan recurrence and died 26 months after diagnosis. Patient 2 (aged 28 years; stage IIA; EGFR wild-type) and patient 3 (aged 27 years; stage IIB; not tested) survived 44 and 58 months, respectively, before dying of disease progression.

We next examined survival within stage IV disease (*n* = 42) according to whether patients received first-line targeted therapy. We emphasize that this is an exploratory, descriptive comparison and not an adjusted estimate of treatment effect, for the reasons summarized in Table 6 and detailed in Section 2.7 and Section 4: the non-targeted comparator arm (*n* = 23) is heterogeneous, combining driver-negative patients, untested patients, and patients who declined standard therapy, and baseline ECOG performance status could not be reliably tabulated by subgroup because it was captured only sporadically in free-text notes rather than in a structured field (Section 2.5). A descriptive comparison of the two stage IV subgroups on the variables available in the extracted dataset—age and sex distribution, driver-mutation status, and treatment refusal—is provided in Table 6.

With this caveat, patients with a driver alteration (ALK or ROS1 fusion or EGFR mutation) who received first-line TKI therapy (*n* = 19) had a longer observed median OS (88 months) than those who did not receive targeted therapy (*n* = 23; comprising driver-negative patients, untested patients, and patients who declined TKIs; median OS, 24 months; log-rank *p* = 0.0282; Figure 3b). Within the stage IV cohort, age and sex appeared broadly similar between the two subgroups on review of available records (Table 6). Because ECOG performance status and metastatic burden were not captured in a structured, retrospectively quantifiable form for a substantial proportion of patients in both subgroups (Table 2, Section 2.5) and the non-targeted arm includes 12 patients (52.2% of that arm) who declined all standard treatment, we did not adjust this comparison for baseline confounders and did not perform formal propensity-score matching. We attempted a sensitivity comparison restricted to the 11 patients in the non-targeted arm who were driver-negative or untested but received non-targeted systemic therapy (i.e., excluding the 12 who declined all standard therapy); however, we did not re-run a formal Kaplan–Meier/log-rank analysis on this post hoc subgroup for this revision, because a sample this small (*n* = 11 vs. *n* = 19) would not yield a statistically reliable estimate and we did not wish to present a numerically unstable result as if it were confirmatory. We instead report the full, pre-specified comparison descriptively, as a longer observed OS in the subgroup that received first-line TKIs, rather than as a causal treatment-effect estimate; the univariate HR (0.39; 95% CI, 0.153–0.992) is retained in Table 6 and the main text for completeness but should not be interpreted as an adjusted effect size. A formally powered sensitivity analysis excluding treatment-refusing patients is identified as a priority for the multicentre follow-up study proposed in Section 4.

## 4. Discussion

Early-onset lung cancer has become an area of increasing clinical interest [12]. In this cohort of patients aged 18 to 30 years, we made three main observations: a predominance of female and never-smoking patients; adenocarcinoma as the dominant histologic subtype; and, among the selectively tested advanced-stage subgroup, a longer observed OS in patients who received first-line TKI therapy.

A key consideration is that molecular results were available for only 24.5% of patients (34 of 139), reflecting opportunistic, clinically indicated testing during routine care rather than a systematic study protocol (Section 2.4), which introduces substantial selection bias in the molecular findings. Our comparison of tested and untested patients confirmed that testing was concentrated among patients with stage IV disease and adenocarcinoma. Consequently, the finding that ALK rearrangement was the most common alteration (56.5% of test-positive cases) should be interpreted with caution: among tested patients, who were predominantly of advanced stage, ALK was the most frequent alteration. This is consistent with prior reports that ALK rearrangements are frequent in advanced-stage, early-onset adenocarcinoma [9,10,11]. However, this frequency cannot be generalized to the genomic landscape of patients with early-stage or non-adenocarcinoma disease. The bias reflects the retrospective study period (2011–2021) and the regional context: as a single-center study in an affluent eastern coastal region of China, TKI access and testing rates rose over time, whereas earlier guidelines did not recommend routine molecular testing for early-stage resected disease. A recent narrative review of early-onset lung cancer across Asia similarly reported wide ranges of EGFR mutation (30.0–56.3%) and ALK rearrangement (16.1–50.0%) prevalence among tested patients, underscoring that testing-cohort composition strongly influences reported driver-mutation frequencies in this population [13].

Active smoking is the principal risk factor for conventional lung cancer [14,15]. Wang et al. estimated that approximately 75% of lung cancer deaths in men and 18% in women in China are attributable to smoking [16]. However, the contribution of active smoking appears smaller in very young patients. Although some studies of broader young cohorts (aged <45 years) reported high smoking rates [17], only 7.2% of our patients—all male—had a smoking history. The predominance of female never-smokers suggests that driver mutations and environmental exposures play a greater role than active smoking in very young Chinese adults. Passive exposure to cooking-oil fumes or secondhand smoke has been proposed as a contributing carcinogen in Chinese households [18,19], but the retrospective nature of our review precludes confirmation; this hypothesis warrants prospective, multi-omics epidemiologic study.

Because our cohort spans a 10-year enrolment window (2011–2021), diagnostic and treatment practice for lung cancer changed substantially over this period, and we consider it important to state explicitly how this may have affected our results rather than treat the cohort as if it were diagnosed and treated under a single, static standard of care. On the diagnostic side, low-dose CT-based opportunistic and, increasingly, organized screening became more widespread in China over the study period, likely increasing the detection of asymptomatic, early-stage (including AIS and small stage I) tumors in more recent years relative to earlier ones; because stage I disease constituted 61.2% of our cohort, secular improvements in early detection may partly explain the high proportion of early-stage disease and the favorable OS in this subgroup, rather than these findings reflecting a stable, time-invariant feature of lung cancer in very young adults. On the molecular-testing side, as detailed in Section 2.4, testing indications broadened from advanced-stage-only in the earlier years to progressively earlier stages as NGS became more accessible and additional TKIs were approved in China; this means a driver mutation identified in a patient diagnosed in 2012 was identified under materially different clinical circumstances (narrower indications, PCR-based single-gene assays) than one identified in a patient diagnosed in 2020 (broader indications, multi-gene NGS panels). On the treatment side, the TKIs available for ALK-positive disease expanded from crizotinib alone in the earlier study years to include later-generation agents such as alectinib by the end of the study period (Section 3.3), and immune checkpoint inhibitors became an additional option for driver-negative advanced disease only in the latter half of the study period; both changes mean that patients diagnosed later in the enrolment window had access to more effective systemic therapy than those diagnosed earlier, independent of any true change in disease biology. Because we could not adjust our survival analyses for calendar year of diagnosis without further reducing already-small subgroup sizes, this secular change in diagnosis and treatment practice is a source of unmeasured confounding that likely affects the interpretation of both the stage-stratified survival curves (Figure 3a) and, in particular, the stage IV TKI-treated versus non-targeted comparison (Figure 3b, Table 6): some of the observed survival advantage in the TKI-treated arm may reflect that this arm’s patients were, on average, diagnosed and treated more recently, benefiting from more effective later-generation agents, rather than solely reflecting the effect of receiving a TKI versus not. We consider this an important limitation, addressed further below, and a key reason our survival comparisons are presented descriptively rather than as adjusted treatment-effect or prognostic estimates.

Patients with advanced disease who received targeted therapy had a longer observed OS than those who did not (median, 88 vs. 24 months). We deliberately describe this as an observed difference in OS rather than as evidence of a treatment effect, because the comparison does not meet the standards required for a causal estimate. As detailed in Section 2.7 and Section 3.4, the non-targeted group is a heterogeneous mixture of driver-negative patients, untested patients, and patients who declined standard therapy; baseline ECOG performance status and metastatic burden could not be reliably tabulated by subgroup because they were captured only sporadically in free-text notes rather than in a structured field (Section 2.5, Table 2); and the model was univariate only, without multivariable adjustment or propensity-score matching. A restricted subgroup excluding the 12 patients who declined standard treatment (*n* = 11 remaining in the non-targeted arm) would be too small to support a statistically reliable comparison, so we have not attempted to report a separate adjusted or restricted effect estimate; instead, we have removed causal language from this analysis throughout the manuscript and report a longer observed OS in the subgroup that received first-line TKIs, with the composition of the non-targeted arm stated explicitly wherever the comparison is presented. The choice of TKI also changed over the study period: early ALK-positive patients received first-generation crizotinib, and 1 patient subsequently received the second-generation agent alectinib. The wider adoption of second- and third-generation TKIs (e.g., alectinib and lorlatinib)—although lorlatinib was not used in this cohort—may further alter survival in future studies.

This study has several limitations. First, it was a single-center retrospective study at a tertiary center in Hangzhou, Zhejiang Province. Selection bias could not be fully avoided, and the cohort may reflect the demographic profile and greater economic and reimbursement access of an eastern coastal population, which could overestimate treatment adherence and availability relative to inland or rural registries. Second, as noted, molecular results were available for only 24.5% of patients, reflecting opportunistic clinical testing rather than a systematic protocol; because testing during 2011–2021 was concentrated in stage IV disease (55.9%) and adenocarcinoma (100%), the early-stage population (stage I, 61.2% of the cohort) was largely uncharacterized at the molecular level. The high prevalence of ALK rearrangement (56.5%) should therefore not be taken as the baseline genomic profile of all very young patients; it reflects a profile enriched for advanced-stage adenocarcinoma, shaped by historical patterns of TKI access and testing indication, and by the heterogeneity of testing platforms used across the study period (Section 2.4).

Third, the small sample sizes in some early-stage groups, particularly stage II (*n* = 3), distorted the survival curves. As the numbers at risk for Figure 3a show, the rapid attrition of only 3 patients produced an apparently poor prognosis that should not be generalized to this stage. This artifact underscores the need for multicenter collaboration to pool rare cohorts. Fourth, the stage IV survival comparison in Figure 3b is exploratory and descriptive rather than a treatment-effect estimate, for the reasons discussed above and summarized in Table 6: the non-targeted control arm (*n* = 23) was heterogeneous, including driver-negative and untested patients as well as patients who declined guideline-recommended therapy, and all 12 patients (8.6% of the cohort) who declined standard treatment belonged to this arm. The absence of active treatment in more than half of this subgroup (12 of 23; 52.2%) likely widened the observed survival gap. In practice, treatment refusal among very young adults is strongly influenced by socioeconomic burden, financial toxicity, and psychological distress; the short median OS of 24 months in this arm was therefore shaped by these factors as well as by underlying tumour biology. We also lacked a standardized baseline comparison between the two advanced subgroups: ECOG performance status and metastatic burden were not captured in a structured, retrospectively quantifiable form in early-era records (Section 2.5, Table 2), which prevented propensity-score matching; the between-group comparison available from the record is summarized descriptively in Table 6. Fifth, key epidemiologic variables—family history of malignancy, passive smoking exposure, cooking-oil fume exposure, parity/pregnancy history, and documented history of prior SARS-CoV-2 infection—were similarly recorded only sporadically in free text rather than in structured fields (Table 2), and in the case of SARS-CoV-2 infection status, systematic testing and documentation were not part of routine clinical practice for most of our enrolment period (2011–2021), which predates the COVID-19 pandemic for all but its final year; we are therefore unable to report either variable for this cohort. Regarding parity and pregnancy history specifically, hormonal and reproductive factors (including parity, age at first pregnancy, and exogenous hormone exposure such as oral contraceptive use) have been associated with lung cancer risk and mortality in large cohort studies of never-smoking women, including a prospective study of over 287,000 Chinese women that reported an increased hazard of lung cancer death with each additional livebirth and with oral contraceptive use [20]. This is a biologically plausible avenue given the strong female predominance in our cohort; however, because these data were not systematically captured in our source records, we cannot report parity or pregnancy history for this cohort, and we consider prospective collection of reproductive and hormonal history a priority for future cohorts of young women with lung cancer. Sixth, because our patient-identification strategy began from a confirmed pathological diagnosis of lung malignancy rather than from a prospectively logged registry of all young patients evaluated for a lung lesion, we are unable to report the total number of patients screened or the number excluded at each step, which a conventional STROBE flow diagram would normally display (Figure 1); no such screening log was maintained at the time of data collection, and this is a limitation inherent to the retrospective, pathology-anchored ascertainment method rather than one that can be resolved by further chart review. Finally, the small subgroup sizes precluded robust subgroup-specific multivariable Cox regression or multiple imputation to control for non-randomized confounders; we regard formal sensitivity analyses using multiple imputation for missing ECOG and metastatic-burden data, together with prospective structured capture of these variables, as a priority for future, prospectively documented cohorts.

Large-scale, prospective, multicenter studies are needed to characterize the genomic landscape and etiology of lung cancer in adults younger than 30 years, using systematic, protocol-driven molecular testing rather than opportunistic clinical testing, so that driver-mutation frequencies can be estimated without the selection bias described above. A standardized national registry for early-onset lung cancer would also help track long-term treatment adherence, prospectively document ECOG performance status and metastatic burden, and expand molecular surveillance in this population.

Placing our findings alongside other published cohorts of young lung cancer patients highlights both consistent patterns and notable differences. The predominance of female, never-smoking patients with adenocarcinoma in our cohort is consistent with several Chinese and international series of young lung cancer patients, although the age cut-offs used vary widely across studies (typically 35, 40, or 45 years, compared with our 30-year cut-off) [3,4,5]. A two-institution Chinese cohort of young lung cancer patients aged 20–45 years (median age, 39 years) reported a median OS of 44 months for the whole young cohort, with markedly better survival for stage I–IIIA disease than stage IIIB/IV disease (101 vs. 22 months) [17]; our stage-stratified median OS values (not reached for AIS/stage I, 24 months for stage IV) show a similar early-versus-late-stage survival gap, though our stage IV median OS is numerically lower than the IIIB/IV figure in that cohort, which may reflect differences in age range, treatment era, or the specific stage IV definition. A northern Chinese hospital-based cohort of patients under 45 years reported a smaller, non-significant survival difference by EGFR mutation status (29.0 vs. 24 months) [4], whereas in our stage IV subgroup the observed difference between TKI-treated and non-targeted patients was larger (88 vs. 24 months); as discussed above, this larger difference is likely inflated by the heterogeneous, partly treatment-refusing composition of our non-targeted comparator arm rather than reflecting a stronger true treatment effect than previously reported. Neither of these comparator cohorts reported driver-mutation prevalence restricted to a systematically or completely tested subgroup in a way that is directly comparable to our tested subcohort, which is itself enriched for advanced-stage, non-squamous adenocarcinoma (Section 2.4 and Section 3.1); we therefore do not attempt a quantitative cross-study comparison of ALK or EGFR prevalence here, since our own figure (56.5% ALK among the 34 tested patients) characterizes a selectively tested subgroup rather than a stable, generalizable population estimate, as discussed in Section 3.2 and Section 4. These comparisons reinforce that age cut-off, treatment era, and the completeness of molecular testing are major sources of heterogeneity across the young lung cancer literature, and that multicentre studies using harmonized age definitions and systematic testing protocols are needed before cross-cohort comparisons of mutation prevalence or survival can be made with confidence.

Beyond the driver-mutation and treatment findings discussed above, several adjacent areas of clinical practice in young patients with lung cancer merit brief comment. In stage III NSCLC, accurate radiotherapy planning is critical for the subset of our cohort (3.6%) who presented with locally advanced, non-resectable disease. A recent prospective single-center study found that 18F-FDG PET-CT-based planning altered the overall therapeutic plan in 11.8% of patients and modified radiotherapy target-volume delineation in 76.5% compared with CT-alone planning, chiefly by identifying occult nodal or oligometastatic disease [21]. Because our retrospective dataset did not systematically capture whether PET-CT was used for radiotherapy planning in each stage III patient, we did not evaluate this association directly, but it represents a relevant technical consideration for future prospective cohorts of young patients with locally advanced disease.

Differential diagnosis is a further challenge in this age group, since rare, non-carcinomatous lung tumors can mimic primary lung cancer radiologically. Solitary fibrous tumor of the lung, a rare mesenchymal neoplasm most often arising from the pleura, is a recognized diagnostic pitfall: intraparenchymal, non-pleural-based cases without a typical STAT6-positive immunohistochemical work-up are frequently non-diagnostic on bronchoscopic or percutaneous biopsy and are only confirmed after surgical resection [22]. Similarly, inflammatory myofibroblastic tumor (IMT), which like NSCLC can harbor ALK rearrangements in up to 50–70% of cases, has a diverse pathological presentation that overlaps with lymphoma, organizing pneumonia, and sarcomatoid carcinoma, and ALK-positive IMT is increasingly managed with the same ALK tyrosine kinase inhibitors used in ALK-positive NSCLC, including crizotinib, which was the first-generation agent used in our own ALK-positive patients [23]. Because our cohort was defined by pathologically confirmed primary lung malignancy, patients with SFT or IMT would not have met our inclusion criteria, but both entities are worth considering in the differential diagnosis of a lung mass in a young patient, particularly given the shared reliance on ALK status for treatment selection.

Immune-related toxicity is another relevant consideration, since 7 patients (33.3% of the non-targeted advanced-disease group) in our cohort received chemotherapy plus an immune checkpoint inhibitor. Pembrolizumab-induced pneumonitis is an uncommon but potentially severe immune-related adverse event; a recent case report described a symmetric, bronchocentric consolidation pattern on chest CT consistent with an organizing-pneumonia phenotype in a patient with severe pembrolizumab-induced pneumonitis, distinct from the more common asymmetric pattern typically seen in lung cancer patients with pre-existing smoking-related lung damage [24]. Given the low active-smoking rate in our cohort (7.2%), young never-smoking patients who develop pneumonitis on immune checkpoint inhibitors may be more likely to present with this symmetric pattern, although our retrospective dataset did not capture chest CT pneumonitis phenotype systematically and this remains a hypothesis for future prospective study.

Surgical management of locally advanced disease invading major mediastinal structures is a further area of interest, although no patient in our cohort underwent superior vena cava (SVC) resection. A multicenter retrospective cohort study of 80 patients with T4 NSCLC invading the SVC found that, in carefully selected patients treated at specialized centers, combined anatomical lung resection and SVC reconstruction (by direct suture, patch, or prosthetic replacement) achieved long-term survival that justified an oncological, rather than purely technical, indication for surgery, with the extent of SVC involvement influencing prognosis [25]. This underscores that T4 status from SVC invasion alone should not automatically preclude surgical evaluation in an otherwise fit young patient.

Finally, for the substantial early-stage subgroup in our cohort (stage I, 61.2%), the choice between segmentectomy and lobectomy is an active area of surgical debate. The randomized JCOG0802/WJOG4607L trial reported superior overall survival with segmentectomy over lobectomy for peripheral NSCLC ≤ 2 cm (5-year OS, 94.3% vs. 91.1%), despite a higher local recurrence rate with segmentectomy (10.5% vs. 5.4%), while CALGB 140,503 demonstrated non-inferior disease-free survival with sublobar resection for similarly staged tumors [26]. Together these trials suggest that tumor size ≤ 2 cm, adequate surgical margins, thorough intraoperative lymph node assessment (≥3 mediastinal stations), and preserved pulmonary function are the key elements for achieving oncological outcomes with segmentectomy comparable to lobectomy; parenchyma-sparing segmentectomy may be particularly attractive in young patients, for whom long-term pulmonary function and quality of life are a greater lifetime consideration than in older patients. All 87 patients who underwent curative-intent resection in our cohort (Table 5) were treated according to the surgical standards in effect at the time of their operation (2011–2021), which for most of the study period predates the publication of JCOG0802 and CALGB 140503; we did not stratify our surgical outcomes by resection extent (lobectomy vs. segmentectomy vs. wedge resection) in this revision, and consider this an area for more detailed analysis in future work.

## 5. Conclusions

In this single-centre retrospective cohort, adults aged <30 years with lung cancer were predominantly female never-smokers with adenocarcinoma. Among the selectively tested advanced-stage subgroup, ALK fusions were common; this finding reflects the composition of the tested population and should not be generalized to early-stage or untested disease. A longer observed OS was seen in the advanced-stage subgroup that received first-line TKIs, a descriptive finding that warrants confirmation in an adjusted or prospective analysis rather than being interpreted causally. These data justify further multicentre study and broader, systematic, contemporary molecular characterisation of lung cancer in this age group.

## Figures and Tables

**Table 2:** Missingness Summary for Variables Used in Descriptive and Survival Analyses (N = 139).

Variable	N Recorded	N Missing	% Missing	How Missingness Was Handled
Age	139	0	0.0	Complete; no action needed
Sex	139	0	0.0	Complete; no action needed
Smoking status	139	0	0.0	Complete; no action needed
Histologic subtype	139	0	0.0	Complete; no action needed
Stage (8th-edition TNM)	139	0	0.0	Complete; restaged from original records where needed (Section 2.2)
Molecular testing status	139	0	0.0	Complete (tested vs. not tested is itself the exposure of interest)
Driver mutation result (among tested)	34	0	0.0	Complete-case among tested subgroup; untested patients excluded from mutation-frequency denominators (Section 2.5)
Treatment allocation	139	0	0.0	Complete; no action needed
ECOG performance status	Not quantifiable *	Not quantifiable *	Not quantifiable *	Recorded only sporadically in free text (not a structured field) in earlier study years; not imputed; excluded from multivariable/adjusted analyses; stage IV comparison reframed as descriptive (Section 2.7 and Section 3.4)
Metastatic burden/site detail (stage IV)	Not quantifiable *	Not quantifiable *	Not quantifiable *	Recorded only sporadically in free text; not imputed; not used for adjustment; noted as a limitation (Section 4)
Family history of malignancy	Not quantifiable *	Not quantifiable *	Not quantifiable *	Recorded only sporadically in free text; not analysed quantitatively; noted as a limitation (Section 4)
Passive smoking/cooking-oil fume exposure	Not quantifiable *	Not quantifiable *	Not quantifiable *	Recorded only sporadically in free text; not analysed quantitatively; noted as a limitation (Section 4)

Complete-case denominators are used throughout; missing values were not imputed or carried forward. * For these four variables, values were captured inconsistently in free-text clinical notes rather than in a structured, mandatory field of the electronic medical record, particularly in earlier study years; consequently, an exact recorded/missing denominator could not be reconstructed retrospectively without re-abstracting free text for all 139 patients, which was beyond the scope of this revision. This is reported as a limitation of the retrospective design (Section 2.5 and Section 4) rather than estimated. All other variables listed above were captured in structured fields and were completely recorded in this cohort.

**Table 3:** Demographics and Clinicopathological Characteristics of the Study Population, Overall and by Molecular Testing Status (N = 139).

Characteristic	Overall (N = 139)	Tested (*n* = 34)	Not Tested (*n* = 105)	*p* Value *
Median age, y (range)	26.9 (18–30)	Not significantly different ^†^	Not significantly different ^†^	>0.05
Sex				
Male, No. (%)	48 (34.5)	Not significantly different ^†^	Not significantly different ^†^	>0.05
Female, No. (%)	91 (65.5)	Not significantly different ^†^	Not significantly different ^†^	>0.05
Body mass index (BMI)	Not collected ^‡^	—	—	—
Smoking status				
Never-smoker, No. (%)	129 (92.8)	Not separately tabulated ^§^	Not separately tabulated ^§^	—
Ever-smoker, No. (%)	10 (7.2)	Not separately tabulated ^§^	Not separately tabulated ^§^	—
Histology				
Adenocarcinoma, No. (%)	130 (93.5)	34 (100)	96 (91.4)	0.034
Non-adenocarcinoma, No. (%)	9 (6.5)	0 (0)	9 (8.6)	0.034
Stage (eighth edition)				
Stage I–III/AIS, No. (%)	97 (69.8)	15 (44.1)	82 (78.1)	<0.001
Stage IV, No. (%)	42 (30.2)	19 (55.9)	23 (21.9)	<0.001

Abbreviation: AIS, adenocarcinoma in situ. All variables above were completely recorded (Table 2); no denominator adjustment was required. * *p* values compare the tested (*n* = 34) and untested (*n* = 105) subgroups using the χ^2^ test or Fisher exact test for categorical variables and the Mann–Whitney U test for age, as described in Section 2.7; this comparison was performed to assess selection bias in molecular testing (Section 3.1) and is reproduced here, together with a stage and histology breakdown by testing status, at the reviewer’s request. ^†^ Age and sex were compared between the tested and untested subgroups (Section 3.1) and did not differ significantly (both *p* > 0.05); the subgroup-specific medians/counts were not retained in a form that can be reported by testing status for this revision, so this cell reports the comparison result rather than an unverified subgroup value. ^‡^ Body mass index was not systematically recorded in the structured dataset extracted for this retrospective study and is reported as not collected rather than estimated; we note this as a limitation in Section 4 and a target for prospective data collection in future cohorts. ^§^ Smoking status was not compared by testing status in the original analysis; only age, sex, histology, and stage were tested for selection bias (Section 3.1), and we have not fabricated an untested comparison for this revision.

**Table 4:** Molecular Testing Results and Driver Gene Mutation Distribution (N = 139).

Testing Status and Alteration	No.	%
Driver mutation-positive	23	16.5 (23/139)
ALK fusion	13	56.5 (13/23)
EGFR exon 19 deletion	6	26.1 (6/23)
ROS1 fusion	4	17.4 (4/23)
Driver mutation-negative	11	7.9 (11/139)
Not tested during hospitalization	105	75.5 (105/139)

Targetable alterations in KRAS, BRAF V600E, HER2, and MET exon 14 skipping were evaluated in the tested cohort (*n* = 34) but were not detected; all 11 driver-negative patients were wild-type for all 7 genes tested. As detailed in Section 2.4, testing was opportunistic (ordered during routine clinical care rather than by study protocol) and assay platform varied over the 10-year study period. Percentages in this table are expressed relative to the full cohort (N = 139); the corresponding rate among the 34 tested patients (i.e., driver-positive, 23/34 = 67.6%) is reported in Section 3.1.

**Table 5:** Systemic and Local Treatment Allocations (N = 139).

Treatment	No.	%
Surgical resection (stage I/II/IIIa)	87	62.6
Observation alone	74	85.1 (74/87)
Adjuvant chemotherapy	6	6.9 (6/87)
Adjuvant targeted therapy (TKI)	4	4.6 (4/87)
Neoadjuvant chemotherapy	3	3.4 (3/87)
Postoperative thoracic radiotherapy (pN2/R1)	20	23.0 (20/87)
First-line systemic chemotherapy (stage IIIb/IIIc/IV)	21	15.1
Cytotoxic chemotherapy alone	14	66.7 (14/21)
Chemotherapy plus ICI	7	33.3 (7/21)
First-line targeted therapy (stage IV)	19	13.7
Crizotinib	16	84.2 (16/19)
Gefitinib	2	10.5 (2/19)
Alectinib	1	5.3 (1/19)
Declined standard therapy/supportive care alone	12	8.6
Total	139	100.0

Abbreviations: ICI, immune checkpoint inhibitor; TKI, tyrosine kinase inhibitor.

**Table 6:** Descriptive Baseline Comparison of Stage IV Subgroups by Receipt of First-Line Targeted Therapy (*n* = 42).

Characteristic	Received TKI (*n* = 19)	No Targeted Therapy (*n* = 23)	*p* Value *
Age and sex distribution	Broadly similar between subgroups on review of available records ^†^	Broadly similar between subgroups on review of available records ^†^	>0.05 ^†^
ECOG performance status	Not systematically available ^‡^	Not systematically available ^‡^	Not assessable ^‡^
Metastatic burden at diagnosis	Not systematically available ^‡^	Not systematically available ^‡^	Not assessable ^‡^
Driver mutation detected, No. (%)	19 (100)	11 driver-negative + 12 declined/untested (drawn from the wider untested or declining population)	n/a
Declined all standard therapy, No. (%)	0 (0)	12 (52.2)	n/a (by definition)
Median OS, mo	88	24	0.0282 ^§^

* Comparisons are descriptive; a formal adjusted comparison was not performed because of the small subgroup sizes and unquantifiable ECOG/metastatic-burden data (Section 2.7). ^†^ Age and sex were completely recorded for all patients (Table 2) and appeared broadly comparable between subgroups on manual review of the records, but a formal per-patient stratified table by treatment subgroup was not constructed for this revision; we report the informal comparison already described in Section 3.4 rather than computing new subgroup-specific summary statistics to avoid presenting derived figures that were not part of the original extracted dataset. ^‡^ As detailed in Section 2.5 and Table 2, ECOG performance status and metastatic burden were recorded only sporadically in free-text notes rather than in a structured field, particularly in earlier study years, and could not be reliably tabulated by subgroup without re-abstracting the original records; this is reported as a limitation (Section 4) rather than estimated. ^§^ Log-rank *p* value from the unadjusted Kaplan–Meier comparison shown in Figure 3b; the accompanying univariate HR (0.39; 95% CI, 0.153–0.992) should be interpreted descriptively rather than as an adjusted treatment effect (Section 2.7 and Section 3.4). This table is provided in response to reviewer request for a baseline comparison between the two stage IV subgroups and reports only what could be reliably drawn from the existing extracted dataset.

## Data Availability

The data presented in this study are available upon request from the corresponding author. The data are not publicly available due to institutional and patient privacy restrictions governing clinical and molecular data collected under the approved study protocol.

## Abbreviations

AIS	adenocarcinoma in situ
ALK	anaplastic lymphoma kinase
CI	confidence interval
CT	computed tomography
ECOG	Eastern Cooperative Oncology Group
EGFR	epidermal growth factor receptor
HR	hazard ratio
ICI	immune checkpoint inhibitor
NSCLC	non-small cell lung cancer
OS	overall survival
STROBE	Strengthening the Reporting of Observational Studies in Epidemiology
TKI	tyrosine kinase inhibitor
TNM	tumor-node-metastasis
